# A case report of disconnected pancreatic duct syndrome: an underrecognized complication of acute necrotizing pancreatitis

**DOI:** 10.1093/omcr/omaf101

**Published:** 2025-07-14

**Authors:** Oumaima Mesbah, Kaoutar Imrani, Safae Lanjeri, Khaoula Boumeriem, Nabil Mouatassim Billah, Ittimade Nassar

**Affiliations:** Department of Radiology, Ibn Sina University Hospital, Faculty of Medicine and Pharmacy of Rabat, Mohamed V University, Souissi Rabat 10000, Morocco; Department of Radiology, Ibn Sina University Hospital, Faculty of Medicine and Pharmacy of Rabat, Mohamed V University, Souissi Rabat 10000, Morocco; Department of Radiology, Ibn Sina University Hospital, Faculty of Medicine and Pharmacy of Rabat, Mohamed V University, Souissi Rabat 10000, Morocco; Department of Radiology, Ibn Sina University Hospital, Faculty of Medicine and Pharmacy of Rabat, Mohamed V University, Souissi Rabat 10000, Morocco; Department of Radiology, Ibn Sina University Hospital, Faculty of Medicine and Pharmacy of Rabat, Mohamed V University, Souissi Rabat 10000, Morocco; Department of Radiology, Ibn Sina University Hospital, Faculty of Medicine and Pharmacy of Rabat, Mohamed V University, Souissi Rabat 10000, Morocco

**Keywords:** acute necrotizing pancreatitis, pancreatic duct disconnection, disconnected pancreatic duct syndrome, walled-off necrosis, pancreatic pseudocyst

## Abstract

Disconnected pancreatic duct syndrome (DPDS) is a rare condition characterized by a disruption of the pancreatic duct, separating viable pancreatic tissue from the gastrointestinal tract. It often follows acute or chronic pancreatitis, abdominal trauma, or pancreatic surgery, leading to ductal necrosis or disintegration. DPDS presents significant diagnostic and management challenges, especially in cases with delayed onset. The authors report a complex case of recurrent pancreatic fluid collections after necrotizing pancreatitis, highlighting the potential for delayed DPDS manifestation. Advanced imaging techniques, including endoscopic ultrasonography, contrast-enhanced CT, and MRCP, were used for diagnosis. Due to failure of conservative treatment, a distal pancreatectomy was performed, resolving the issue and preventing complications such as infection, sepsis, or pancreatic fistula. This case underscores the importance of early recognition of DPDS on imaging, facilitating timely treatment and reducing the risk of long-term complications.

## Introduction

The term ‘disconnected pancreatic duct’ (DPD) refers to an anatomical condition where the ductal continuity between the healthy pancreatic tissue and the duodenum is interrupted. This can occur in acute necrotizing pancreatitis when a segment of the main pancreatic duct becomes necrotic, leading to a separation between the pancreas and the duodenum [1]. Despite the disruption, the affected segment may continue to secrete pancreatic fluids, which can cause ongoing external pancreatic fistulas, recurrent fluid collections, or obstructive recurrent acute or chronic pancreatitis in the isolated part of the pancreas. This condition is often missed or diagnosed late in clinical practice, but identifying it is crucial for proper therapeutic decision-making.

The global burden and incidence of Disconnected Pancreatic Duct Syndrome (DPDS) are not well-defined due to its rare occurrence and diagnostic challenges. The few available reports in the literature suggest a range between 16% and 50%, but this incidence refers specifically to high-risk groups such as patients with necrotizing pancreatitis, walled-off pancreatic necrosis (WON), pancreatic trauma, or post-surgical complications, where the main pancreatic duct is more prone to disconnection due to necrosis or injury [2]. However, the true incidence might be higher since many cases are asymptomatic, undiagnosed, and underreported. Diagnosis primarily relies on imaging techniques, including contrast-enhanced computed tomography, endoscopic retrograde cholangiopancreatography, magnetic resonance cholangiopancreatography, and endoscopic ultrasonography [3].

## Case report

A 68-year old female presented to the emergency with complaints of sharp epigastric pain, which worsened over several hours. The pain was described as deep and radiating to the back, becoming more intense after meals, especially fatty foods, occurring within 30–60 min after eating. The pain was rated 8 out of 10 in severity, and the patient found relief by assuming a fetal position. It was constant and unrelenting, exacerbated by movement or deep breathing, and accompanied by nausea and vomiting. She had a history of gallstone acute necrotizing pancreatitis 2 months prior for which she was hospitalized and received a total parenteral nutrition. A cholecystectomy was performed via laparoscopy after the acute episode has resolved and the patient’s condition has stabilized. No other risk factors were identified. At the time of the presentation, the patient showed stable vitals with regular pulse, tachycardia and a normal blood pressure of 120/80 mm/Hg. She was conscious and well oriented.

**Figure 1:**
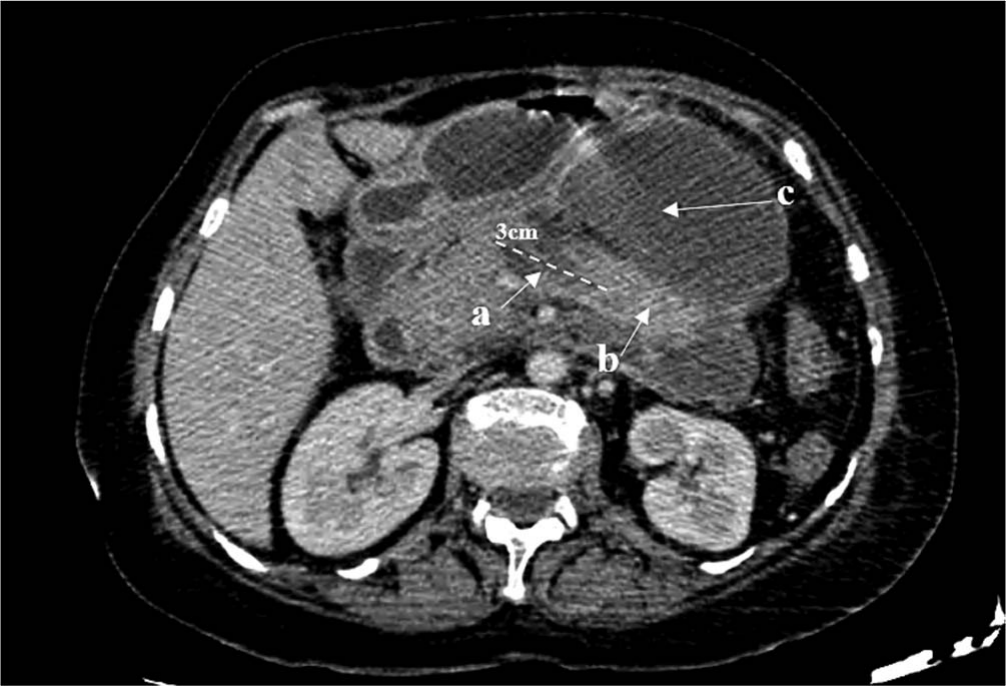
Axial CT scan showing a fluid collection occupying the body (a), measuring 3 cm, with a viable upstream segment (b). Multiple peripancreatic walled-off necrosis (c).

**Figure 2:**
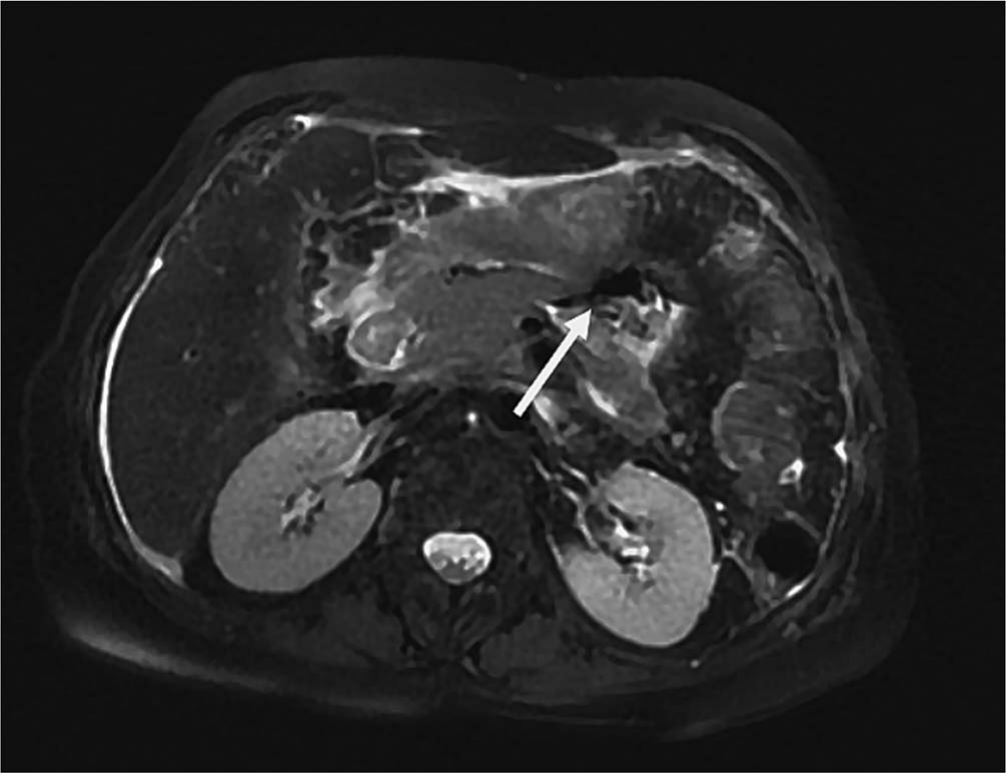
MRI of the abdomen, an axial section, on T2 fat saturated sequence, showing a persistent collection (arrow) in the pancreatic body, as a hyperintense signal and internal T2 hypointense debris, communicating with the main pancreatic duct, measuring 3 cm. Gas bubbles are present in the fluid collection due to a connection with the gastric lumen.Peripancreatic fat stranding is seen.

**Figure 3:**
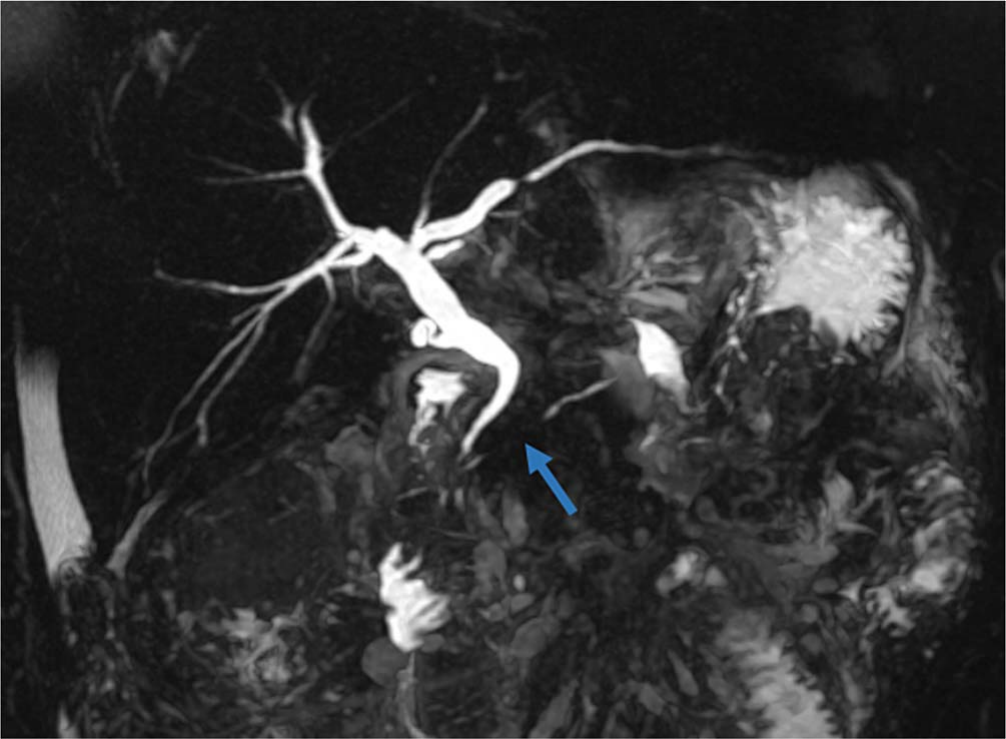
3D oblique MRCP image shows a loss of normal continuity of the main pancreatic duct. Note a dilated main bile duct.

On physical examination, the patient exhibited severe tenderness in the epigastric region, with signs of peritoneal irritation. The abdomen was soft but distended, and palpation of the upper abdomen elicited significant rebound tenderness. No palpable masses or organomegaly were noted. She did not exhibit Cullen’s sign (bluish discoloration around the umbilicus) or Grey-Turner’s sign (ecchymosis in the flank area), both of which can indicate severe pancreatic or retroperitoneal hemorrhage. On hematological examination, white cell count was elevated −16 × 10^9^/L with elevated lipase levels of 560 U/l and amylase levels of 440 U/l, four times their normal level. A contrast enhanced CT scan revealed large fluid collections occupying the body, measuring 3 cm, with a viable upstream segment, with multiple peripancreatic walled-off necrosis. (Fig. 1) An esophagogastroduodenoscopy showed a cystic formation communicating with an intrapancreatic parenchymal necrosis. A centesis of the cyst was performed with the placement of a gastrocystic stent for drainage. A magnetic resonance cholangiopancreatography was performed in the following month due to the persistence of abdominal pain. The MRI of the abdomen shows a persistent collection in the pancreatic body extending into the adjacent peripancreatic space, (Fig. 2) 3D oblique MRCP image shows communication of the main pancreatic duct (MPD) with the corporeal collection with loss of normal continuity of the duct. A viable pancreatic tissue upstream is noted and gas bubbles are present in the fluid collection due to a connection with the gastric lumen. (Fig. 3) Features were suggestive of disconnected pancreatic duct syndrome. Although most peripancreatic fluid collections following acute pancreatitis are managed conservatively or with guided drainage, these methods are typically ineffective in addressing the underlying ductal disconnection.

In our case, due to the failure of the endoscopic drainage by the gastrocystic stent and to prevent further complications such as sepsis and hemorrhage, distal pancreatectomy, removing the viable disconnected pancreatic segment, was performed. Its inability to properly redirect pancreatic secretions into the gastric lumen wasn’t clearly pinpointed, but it can be attributed to a combination of anatomical, procedural, and technical issues. Stent displacement due to the surrounding inflammation or changes in intra-abdominal pressure, obstruction by pancreatic debris or inflammatory tissue, impaired gastric motility and ongoing pancreatic ductal leaks are among the leading causes.

In the postoperative course, the patient was closely monitored for signs of infection, bleeding, and pain. Intravenous fluids and pain management were initiated, and a drain was placed near the pancreatic stump to prevent complications. Postoperative CT scan was performed which confirmed the resolution of peripancreatic collections. The patient made a recovery and was discharged after 8 days. Regular follow-up was recommended to monitor for signs of endocrine and pancreatic insufficiency and follow up imaging was recommended to ensure ongoing resolution.

## Discussion

Disconnected pancreatic duct syndrome is a condition where the continuity of the pancreatic duct between functional distal pancreatic tissues and the gastrointestinal tract is lost. This disruption leads to leakage of pancreatic fluids into surrounding extra-ductal pancreatic tissue, resulting in autolysis of the pancreatic parenchyma at the site of leakage [3, 4]. The condition can be either complete or partial, with its severity varying accordingly. A complete disconnection refers to a total circumferential interruption of the duct integrity, while a duct leak indicates a partial interruption [5]. Distinguishing between these two types is crucial as they require different management strategies.

The pathophysiology of disconnected pancreatic duct syndrome usually stems from acute pancreatitis, though it can also be caused by blunt abdominal trauma, pancreatic surgery, chronic pancreatitis, or pancreatic malignancy. In cases of acute pancreatitis, the syndrome is most commonly seen in patients with severe or necrotizing forms [6, 7].

Ductal integrity is compromised when pancreatic necrosis affects the ductal epithelial cells [8]. These injuries to the main pancreatic duct typically occur in the pancreatic head and the nearby neck and body regions, largely due to the susceptibility of these areas to ischemic necrosis from their fragile vascular supply [9]. In a retrospective study, the pancreatic neck was involved in 57.8% of cases, followed by the distal body-tail in 23.1%, and the mid body in 19.2% [3].

Persistent pancreatic leak can cause further complications including intra-abdominal sepsis, hemorrhage from peripancreatic blood vessels, and exocrine or endocrine pancreatic insufficiency [6].

The diagnosis is primarily based on imaging. Contrast enhanced computed tomography, magnetic resonance imaging with cholangiopancreatography are currently the most applicable methods, allowing the imaging of the pancreas upstream of the disconnection. in addition to the traditional technique of endoscopic retrograde cholangiopancreatography limited by the demonstration of the termination of the downstream portion of the duct [10].

Patients with pancreatitis undergo serial CT imaging and the diagnosis of disconnected main pancreatic duct is usually overlooked on initial CT images unless the duct is visibly dilated.

The criteria of radiologic studies that have been proven to confidently recognize the diagnostic on imaging suggest that the following features are found [11]:—An area of necrotic pancreas measuring 2 cm or greater (healing can occur with less extensive necrosis);—The presence of viable pancreatic tissue upstream from the site of necrosis.—Extravasation or total cut-off of contrast material injected into the MPD at pancreatography—Pancreatic duct (possibly dilated) entering the collection at an approximately 90° angle (if visible).

Treatment decisions for disconnected pancreatic duct syndrome are currently made at the judgment of the treating clinicians due to the absence of standardized guidelines. Typically, patients with this condition may require multiple advanced procedures and rescue surgeries [12]. While most peripancreatic fluid collections following acute pancreatitis are managed conservatively or with guided drainage, this approach is generally ineffective for treating the disconnection itself. Endoscopic drainage can be beneficial by using indwelling transluminal plastic stents, which may be left in place or replaced with lumen-apposing metal stents to maintain the internal fistula’s patency and redirect pancreatic secretions into the gastrointestinal tract [2]. Surgery results in a high success rate and eventually provides a definite solution [2].

Treatment may include either removing the viable disconnected pancreatic segment or creating a pancreaticojejunostomy to drain it. The choice of surgical approach depends on factors such as the patient’s age, the extent of ongoing retroperitoneal inflammation, the size of the disconnected segment, and the status of the pancreas’s exocrine and endocrine functions [12]. If the upstream duct is of adequate size, a Roux-en-Y pancreaticojejunostomy can be performed to preserve pancreatic function and ensure proper and physiological drainage of pancreatic secretions [13].

Distal pancreatectomy with or without splenectomy may be performed if the disconnected segment is small [3].

## Conclusion

The disconnecting pancreatic duct syndrome is underrecognized complication seen frequently in necrotic pancreatitis therefore it is an important finding for the radiologist to look for and recognize. The alarm bells should ring with non-resolving fluid collections, recurrent bouts of pancreatitis, or patients undergoing ineffective interventions.

The condition is a management challenge in many patients, and a multidisciplinary team including gastroenterologists, pancreatobiliary surgeons, and radiologists should always be involved in decision making.

## References

[1] Vanek P, Urban O, Trikudanathan G. et al. Martin L Freeman: disconnected pancreatic duct syndrome in patients with necrotizing pancreatitis. Surg Open Sci 2023;11:19–25. 10.1016/j.sopen.2022.10.009 36438587 PMC9692037

[2] Maatman TK, Roch AM, Lewellen KA. et al. Disconnected pancreatic duct syndrome: Spectrum of operative management. J Surg Res 2019;247:297–303. 10.1016/j.jss.2019.09.068 31685250

[3] Tann M, Maglinte D, Howard TJ. et al. Disconnected pancreatic duct syndrome: imaging findings and therapeutic implications in 26 surgically corrected patients. J Comput Assist Tomogr 2003;27:577–82. 10.1097/00004728-200307000-00023 12886147

[4] Fischer TD, Gutman DS, Hughes SJ. et al. Disconnected pancreatic duct syndrome: disease classification and management strategies. J Am Coll Surg 2014;219:704–12. 10.1016/j.jamcollsurg.2014.03.055 25065360

[5] Nealon WH, Bhutani M, Riall TS. et al. A unifying concept: pancreatic ductal anatomy both predicts and determines the major complications resulting from pancreatitis. J Am Coll Surg 2009;208:790–9. 10.1016/j.jamcollsurg.2008.12.027 19476839

[6] Chen Y, Jiang Y, Qian W. et al. Endoscopic transpapillary drainage in disconnected pancreatic duct syndrome after acute pancreatitis and trauma: long-term outcomes in 31 patients. BMC Gastroenterol 2019;19:54. 10.1186/s12876-019-0977-1 30991953 PMC6469079

[7] Varadarajulu S, Rana SS, Bhasin DK. Endoscopic therapy for pancreatic duct leaks and disruptions. Gastrointest Endosc Clin N Am 2013;23:863–92. 10.1016/j.giec.2013.06.008 24079795

[8] Jang JW, Kim M-H, Oh D. et al. Factors and outcomes associated with pancreatic duct disruption in patients with acute necrotizing pancreatitis. Pancreatology. 2016;16:958–65. 10.1016/j.pan.2016.09.009 27681504

[9] Bang JY, Wilcox CM, Navaneethan U. et al. Impact of disconnected pancreatic duct syndrome on the endoscopic management of pancreatic fluid collections. Ann Surg 2018;267:561–8. 10.1097/SLA.0000000000002082 27849658

[10] Vanek P, Trikudanathan G, Freeman ML. Diagnosing disconnected pancreatic duct syndrome: many disconnects, few answers. Dig Dis Sci 2021;66:1380–2. 10.1007/s10620-020-06538-2 32794056

[11] Sandrasegaran K, Mark Tann S, Jennings G. et al. Disconnection of the pancreatic duct: an important but overlooked complication of severe acute pancreatitis. Radiographics 2007;27:1389–400. 10.1148/rg.275065163 17848698

[12] Howard TJ, Stonerock CE, Sarkar J. et al. Contemporary treatment strategies for external pancreatic fistulas. Surgery 1998;124:627–33. 10.1067/msy.1998.91267 9780981

[13] Pearson EG, Scaife CL, Mulvihill SJ. et al. Roux-en-Y drainage of a pancreatic fistula for disconnected pancreatic duct syndrome after acute necrotizing pancreatitis. HPB (Oxford) 2012;14:26–31. 10.1111/j.1477-2574.2011.00397.x 22151448 PMC3252988

